# Outcome Measures in Relapsing-Remitting Multiple Sclerosis: Capturing Disability and Disease Progression in Clinical Trials

**DOI:** 10.1155/2014/262350

**Published:** 2014-05-04

**Authors:** Amy M. Lavery, Leonard H. Verhey, Amy T. Waldman

**Affiliations:** ^1^Division of Neurology, Children's Hospital of Philadelphia, Philadelphia, PA 19104, USA; ^2^The Pediatric Demyelinating Disease Program, Program in Neuroscience & Mental Health, The Hospital for Sick Children, University of Toronto, Toronto, ON, Canada M5G 1X8; ^3^Departments of Neurology and Pediatrics, Perelman School of Medicine at the University of Pennsylvania, Philadelphia, PA 19104, USA

## Abstract

Multiple sclerosis (MS) is a chronic inflammatory and neurodegenerative disease that manifests as acute relapses and progressive disability. As a primary endpoint for clinical trials in MS, disability is difficult to both characterize and measure. Furthermore, the recovery from relapses and the rate of disability vary considerably among patients. Given these challenges, investigators have developed and studied the performance of various outcome measures and surrogate endpoints in MS clinical trials. This review defines the outcome measures and surrogate endpoints used to date in MS clinical trials and presents challenges in the design of both adult and pediatric trials.

## 1. Introduction

Multiple sclerosis (MS) is a leading cause of morbidity and disability in young adults. There has been significant research in the development and use of outcome measures and surrogate endpoints for MS clinical trials. Such investigations have been necessary due to the evolution in clinical trial design in MS. Early trials sought to determine whether disease-modifying therapies could alter the number of relapses compared to placebo in patients already diagnosed with relapsing-remitting MS. Clinical trials later evolved to determine whether early treatment could delay the second attack in the relapsing portion of the disease compared to placebo. Current trials of new therapies, including oral medications, are mostly active-arm comparison trials, which require more sensitive metrics to determine efficacy. Alternatively, larger sample sizes may be required which may impact the feasibility of a study. Trials of symptomatic therapies, such as 4-aminopyridine for ambulation, and future trials studying agents for neuroprotection and neurorepair, will require different outcome measures to determine neurologic functional recovery compared to those currently being used in MS studies measuring clinical disease. In addition to their use in clinical trials, outcome measures and surrogate endpoints have been applied to the individual patient to evaluate disease progression and the need for a change in therapy.

MS is a heterogeneous disease with considerable variation in the clinical, radiographic, genetic, pathologic, and biologic features among patients. Such differences can present challenges in clinical trial design. It is not uncommon to have multiple primary and secondary endpoints reflecting the diversity. Furthermore, surrogate endpoints are often used to determine treatment efficacy. This review will define outcome measures and surrogate endpoints, discuss the metrics that have been used to date in MS trials, and present challenges in the design of adult and pediatric MS clinical trials.

## 2. Primary Outcome Measures

**Figure 1:**
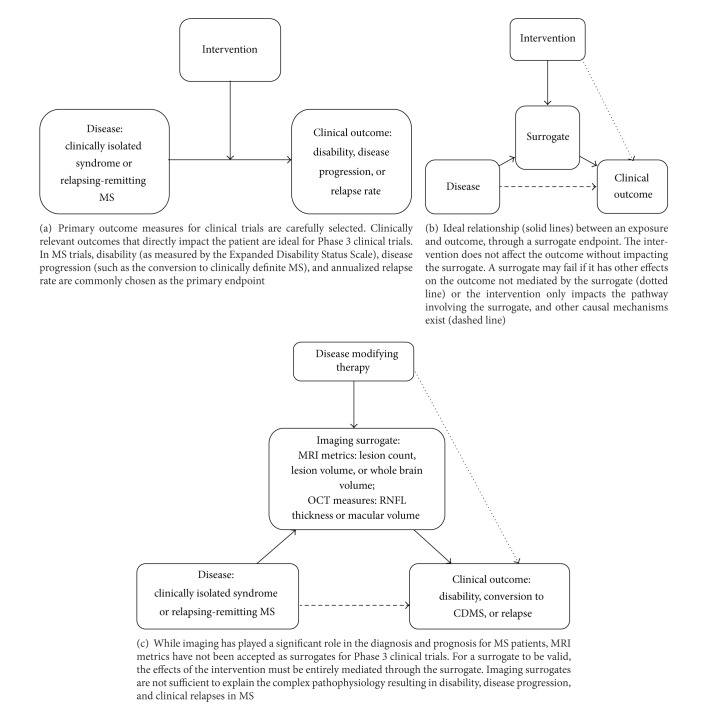
Clinical outcome measures and surrogate endpoints in Phase 3 clinical trials.

Table 1 lists the various adult clinical trials, along with their primary and secondary outcome measures, for relapsing-remitting MS. Selecting an appropriate outcome measure for clinical trials is important in determining whether the intervention is actually modifying the disease course (Figure 1(a)) and evaluating the risk-to-benefit ratio [1]. A measure with poor reliability or interpretability may lead to inaccurate results and improper use of treatments. According to Fleming and DeMets, “For phase 3 trials, the primary end point should be a clinical event relevant to the patient, that is, the event of which the patient is aware and wants to avoid [2].” Disability is a patient-centered outcome that is appropriate for MS trials, akin to survival in cardiac disease or cancer trials.

### 2.1. Quantifying Disability

#### 2.1.1. Expanded Disability Status Scale

While disability may be considered an ideal primary endpoint based on the definition above, disability can be difficult to both define and measure. Patients with MS develop various degrees of visual, cognitive, physical, and psychological disability, and it is difficult, for example, to compare disability related to chronic fatigue affecting one patient to paralysis suffered by another patient. The Expanded Disability Status Scale (EDSS) was created to address this issue and quantify a patient's disability based on scores of eight functional systems [3]. The scale ranges from 0 (normal neurological exam) to 5 (ambulatory without aid for 200 meters) to 10 as the most serious outcome, death due to MS. While this is often a measure used in clinical trials and regular clinical examinations, the scale is not very sensitive to change and lacks a clinically defined relevant change [4].

Clinical trials have defined disability progression as an increase in the EDSS scale of 0.5–1.0 point after 3 or 6 months. Ebers et al. challenge the use of the EDSS, concluding that such change over a short time frame is likely measurement error or random variation and not the result of sustained disability [5]. Consequently, the EDSS may be ineffective at predicting disease progression in RRMS patients after a short time period. The authors propose that longer trials (with a duration of at least 1 year) with greater changes in the EDSS scores (>1-2 points) may better capture patients with sustained disability. This and other studies have shown that the EDSS often has problems with reliability and validity. Interrater variation has been reported to be greater than a 1-point increase in the EDSS about 40% of the time [5, 6]. Despite the limitations of the EDSS, it is accepted as a “true clinical efficacy measure” and has been widely accepted as a primary outcome measure for Phase 3 clinical trials in MS [1].

#### 2.1.2. Multiple Sclerosis Functional Composite

MS researchers recognized the limitations of the EDSS and sought to develop a more responsive clinical outcome measure. The National MS Society Task Force developed the Multiple Sclerosis Functional Composite (MSFC), which uses simple measures to help assess patient functionality [7, 8]. The MSFC consists of a 25-foot timed walk, a 9-hole peg board, and paced auditory serial addition task (PASAT), which reflect ambulation, hand function, and speed of processing. Tests can be administered by nonclinicians and the total time required to take the test is about 15 minutes [9]. A z-score is created based on the average value for each test, allowing for direct comparison of measures with different units [8, 9]. The z-score reports the standard deviation of the individual's performance compared to the mean of the reference population [8].

MSFC scores correlate with EDSS scores. In a study of 300 MS patients, Miller et al. found that the MSFC was highly correlated with the EDSS with a Spearman correlation coefficient of −0.80 [10]. The individual components of the MSFC (arm, leg, and cognitive) also correlate with composite EDSS scores (Spearman rank correlation coefficients −0.33, −0.52, and −0.23, resp.) [8]. Likewise, Rudick et al. determined that changes in MSFC scores (defined as a 15% or 20% change in a single component of the MSFC, sustained for 3 months) correlated with EDSS scores and relapse rates [11].

Furthermore, baseline MSFC scores in clinical trials and change in MSFC score during a 2-year period were shown to predict future disability. Decreased baseline and worsening MSFC scores early in a phase III clinical trial of interferon beta 1a predicted poor outcomes, including physical disability, progression to secondary progressive disease, brain atrophy, and decreased quality of life at a mean of 8.1 years [12].

Similar to the EDSS, however, researchers have indicated difficulty in quantifying a meaningful change in the MSFC components. The tests are also weighted equally in the composite score, which may not accurately capture the disease progression as a whole. Individual components of the test may change over time, and the composite score may not reflect these differences [13]. In a phase II trial of Rituximab as add-on therapy, Naismith et al. found that improvements in MSFC scores were largely attributed to the PASAT [14]. However, improvements in the PASAT may reflect practice effects rather than the true changes in cognitive dysfunction. Also, despite the efforts towards developing the MSFC, it has not been incorporated into many clinical trials as a primary endpoint. Although used in some of the trials listed in Table 1, the MSFC has generally been used as a secondary endpoint along with the EDSS or in further secondary analyses [11, 15].

#### 2.1.3. Low-Contrast Letter Acuity

The MSFC has also been criticized for not including a visual measure; however, at the time of its development, a sensitive test to accurately capture vision loss in MS patients had not been identified. The commonly used Snellen (high-contrast) charts are known to have limited capacity to measure small changes in visual dysfunction [16]. Over the past 10 years, cross-sectional and longitudinal studies of adult MS patients and disease-free controls have demonstrated that low-contrast letter acuity (LCLA) is the most sensitive test for identifying visual dysfunction in patients with MS [4, 17–19] and have been proposed as the visual component of the MSFC [13]. As such, LCLA was performed in the AFFIRM and SENTINEL trials [15, 20, 21]. While high-contrast testing did not differ between study groups, visual function measured using LCLA worsened (defined as a 2-line or 10-letter worsening of visual acuity) over a two-year period in the placebo group. Moreover, Balcer et al. also demonstrated a decreased risk of sustained visual loss in the natalizumab group compared to placebo (a risk reduction of 47% for 2.5% LCLA, *P* < 0.001; and 35% for 1.25% LCLA, *P* = 0.008 for 1.25% LCLA in the AFFIRM trial). The authors concluded that LCLA has the ability to determine treatment effects and proposed that LCLA should be incorporated into future clinical trials [15].

### 2.2. Relapse Rate and Disease Progression

Sustained disability may not be detected early in the disease course for MS patients. Given the additional challenges in quantifying disability, annualized relapse rate (ARR), time to first relapse (TTFR), and conversion to clinically-definite MS (CDMS) have been the most common primary outcome measures used in clinical trials.

Annualized relapse rate is often included as an outcome measure for clinical trials because it is easy to quantify, and prevention of relapses benefits patients immediately. Relapses are generally defined as neurologic symptoms lasting more than 24 hours which occur at least 30 days after the onset of a preceding event [22], though definitions can vary slightly by study. Relapse rate early in the disease is thought to predict future disability [23, 24]. However, the probability of relapses is not a constant function over time. Patients are usually enrolled in a trial at the time of MS diagnosis when the probability for relapses is high, and, as time progresses, this probability decreases due to the regression to the mean phenomenon. In addition, relapses may be separated by several years, which may be very time consuming and costly for a clinical trial. Sormani et al. determined that in order to have enough power to detect a significant reduction in relapses, a clinical trial needs to last at least 1 year, but this measure may also be less meaningful than looking at total number of relapses over a longer period of time [25]. They also suggest that due to low relapse rates recorded in recent trials, the sample size required for new studies may not be feasible [25, 26].

Time to first relapse has been recently proposed as a primary endpoint for clinical trials. Time to first relapse is an appealing alternative to annualized relapse rate because it generally requires less study time to reach the trial endpoint, and the first relapse is typically more accurately documented than the subsequent relapses included in the ARR calculation. Additionally, Sormani et al. determined that TTFR may require a lower number of subjects than trials based on the annualized relapse rate [25]. The authors also note that for clinical trials where it may not be ethical to use a placebo for extended periods of time, using TTFR as the primary outcome measure allows patients enrolled in the placebo arm to switch to the active treatment immediately after a relapse occurs (i.e., the study endpoint).

Conversion to CDMS as a trial endpoint is related to TTFR; for patients enrolled into a trial at the time of an incident demyelinating event (i.e., clinically isolated syndrome [CIS]), the first relapse corresponds to the second clinical attack, or confirmation of CDMS. Early trials of disease-modifying therapies used progression to CDMS as a primary outcome measure. The measure was generally defined as the time to a second clinical attack following the first demyelinating event. While the conversion to CDMS is an ideal outcome measure for many clinical trials, it is often costly and time consuming. Furthermore, with varying definitions of a second attack between studies, even slight differences can affect the reproducibility of treatment efficacy. For example, some studies defined a second attack as neurological symptoms that have persisted for a minimum of 24 hours in the absence of fever with objective findings whereas other studies have required neurological symptoms to persist for at least 48 hours and subjective events were permitted [27–29].

## 3. Surrogate Endpoints

The previously-mentioned outcome measures represent patient-centered outcomes that have been used in Phase 3 clinical trials to determine treatment efficacy. Other secondary measures, often surrogate endpoints, have also been included in the same trials. As defined by Temple: “A surrogate endpoint of a clinical trial is a laboratory measurement or a physical sign used as a substitute for a clinically meaningful endpoint that measures directly how a patient feels, functions, or survives [30].” Various MRI parameters have been proposed as surrogates in MS trials.

### 3.1. Magnetic Resonance Imaging

Magnetic resonance imaging has been incorporated into the diagnostic criteria for MS [31] and serves as a routine paraclinical tool to follow disease progression. Consideration of MRI findings also allows the diagnosis of MS to be made earlier than if relying solely on clinical relapses. The next several paragraphs will address the rationale for including MRI metrics in MS clinical trials followed by a general discussion of the challenges when using MRI measures as surrogates for disease activity.

#### 3.1.1. MRI Lesion Counts

Changes in MRI-visible brain lesions reflect changes in the underlying disease pathology and therefore provide theoretical rationale for using MRI lesions as measures of disease activity. MRI lesions are quantified as the number of T1-weighted gadolinium-enhancing lesions, new T2 lesions, or active (i.e., new or enlarging) T2 lesions. The treatment effect on gadolinium-enhancing lesions is highly associated with the treatment effect on active T2 lesions (*R* ^2^ = 0.93) [32], suggesting that either contrast-enhancing or new T2 lesion endpoints are suitable for monitoring MRI activity in MS clinical trials. It should be noted, however, that using gadolinium-enhancing lesions as an outcome requires monthly MRI scans which in turn increases the cost of the trial. All immunomodulatory agents decrease the number of gadolinium-enhancing and new T2 lesions; the degree and rate of their effect are variable and dependent on the drug's mechanism of action (i.e., at the blood-brain barrier for beta-interferons and more centrally-mediated action for glatiramer acetate). Decreased MRI activity represents the earliest treatment effects in clinical trials and therefore has served as an attractive endpoint in clinical trials.

Lesion load measured early in the disease course is associated with future relapses [23, 33], disability accumulation [34, 35], and cognitive deficits [36]. Based on a meta-analysis of 23 clinical trials in relapsing-remitting MS, the association between the treatment effect on relapse rate is strongly correlated (*R* ^2^ = 0.81) with the treatment effect on MRI lesions (i.e., new or active T2 lesions or gadolinium enhancing lesions if monthly scans were acquired) [37]. Other studies have shown that the effect of interferon-beta on MRI lesions mediates 60% of the effect on relapse rate [38] and 57% of the effect on disability progression [39]. Taken together, these findings suggest the potential role of MRI lesions as a surrogate for disability progression and relapse rate in clinical trials, and also that MRI lesion and relapse activity can serve as early indicators of treatment response in regards to disability progression.

#### 3.1.2. “Black Hole” Formation

“Black holes” are nonenhancing hypointense lesions on T1-weighted imaging that are correlated with areas of focal chronic axonal damage and loss on histopathology [40]. Therefore, the evolution of active lesions into T1-hypointense lesions represents irreversible tissue damage, and their accumulation is associated with disability accrual (*r*_*s*_ = 0.46) [41–43]. In a recent multivariable analysis, worsening of EDSS score over 10 years in 58 patients with RRMS was associated with a combination of baseline T1-hypointense lesion count and increasing T1 lesion volume (*r* = 0.61, *P* < 0.001) [44]. Taken together, these findings support the role for a decline in “black hole” formation as a potential marker for neuroprotective effects. In this context, it is noteworthy that a placebo-controlled trial of glatiramer acetate with monthly MRI monitoring demonstrated a significant reduction in “black hole” lesion formation [45].

#### 3.1.3. T2 Lesion Volume

Measuring changes in total T2 lesion volume is another method of evaluating MRI activity. Robust (semi-) automated volumetric analyses rely on the acquisition of MRI scans according to a standardized and carefully quality-monitored MRI protocol. T2 lesion volume has been shown to be reduced in patients with MS receiving teriflunimide compared to placebo [46]. T2 lesion volume is positively correlated with disability measured by EDSS at 2 [47] and 10 years of follow-up [48] and number of relapses after 2 years of follow up [34, 47]. A recent study evaluated MRI correlates of disability in a cohort of 159 patients with relapsing-remitting MS (median EDSS = 4) followed for mean of 26 years from first attack and found that T2 lesion volume was associated with long-term disability and independent of cervical spinal cord atrophy and grey matter atrophy [49]. Similarly, a study of 107 MS patients followed for mean of 20 years from first attack showed that T2 lesion volume correlated with 20-year EDSS (*r*_*s*_ = 0.48–0.67) and MSFC z-score (*r*_*s*_ = −0.5–0.61).

#### 3.1.4. Brain Volume

Brain volume and atrophy measurements correlate with measures of disability [48, 50, 51] and cognitive function [52, 53]. Considering the previously discussed limitations to using EDSS as a primary outcome measure, the validity of brain atrophy as a surrogate marker for disability progression is of interest in studies on clinical trial design for relapsing-remitting MS. A recent meta-analysis of thirteen randomized clinical trials in relapsing-remitting MS showed that the treatment effect on disability progression (i.e., a 3- or 6-month sustained 1-point increase in EDSS) correlated with the treatment effects on brain atrophy (*R* ^2^ = 0.48) and active T2 lesions. (*R* ^2^ = 0.61) [54]. In fact, the authors showed in a multivariable model that 75% of the variance in treatment effect on disability was explained by the combined effect on active T2 lesions and brain atrophy. These findings, if validated in individual patient-based analyses of clinical trial data, support the use of brain atrophy alone or in combination with active T2 lesions as surrogate markers of disability progression in relapsing-remitting MS.

An important practical implication is that trials powered on the outcome of a 50% reduction in MRI lesions [55] and atrophy [56] require ten times fewer subjects than that required for a trial based on a disability endpoint [54, 57]. Recent data has suggested that placebo-controlled trials evaluating brain atrophy (using the SIENA method) would require 32 subjects per arm (80% power) to detect a 50% treatment effect over 2 years [58].

Given that axonal degeneration and loss are now understood as major contributors to disability in MS [59], considerable attention has been given to defining neuroprotective therapeutic strategies that will slow or prevent disability progression. Changes in brain volume in patients with MS reflect the neurodegenerative biology of the disease, and therefore, MRI markers of neuronal damage may represent potential surrogates to neuroprotective therapy response [60]. Moreover, current brain atrophy measurement techniques are suited to multicentered trials, further supporting the potential role of brain volume surrogate markers in phase II trials of neuroprotective agents [21]. Trials of oral fingolimod [61], oral laquinimod [62], and natalizumab [20] reported a favorable effect of treatment on brain volume loss in relapsing-remitting MS patients, compared to placebo. However, some challenges, such as the effects of brain edema, pseudoatrophy from corticosteroids, and ongoing neurodegeneration from injury prior to trial enrollment that challenge establishment of a stable baseline need to be addressed before brain atrophy is considered as a surrogate marker of treatment effect in clinical trials.

#### 3.1.5. MRI Metrics as Surrogate Outcome Measures

While MRI variables (e.g., lesion count, lesion volume, and brain volume) are commonly used in clinical trials to assess treatment efficacy and disease progression, they are not generally recognized as validated surrogate outcome measures. For a surrogate endpoint to be valid for a Phase 3 trial, it must exist within the causal pathway in the absence of other mechanisms of action between the exposure and the outcome, and an intervention must exert its effects on the clinical outcome through the surrogate (Figure 1(b)). If the intervention has other mechanisms of action, the surrogate may fail [2].

As reflected in Table 1, clinical trials in relapsing-remitting MS incorporate multiple MRI outcome measures as surrogates of disease activity. However, there is a poor correlation between MRI activity and relapses as the appearance of new MRI lesions often outnumber clinical relapses. This “clinico-radiological paradox” in MS became apparent when MRI was first used in MS and attempts to correlate T2 lesions (a nonspecific marker of focal brain injury) with EDSS revealed a dissociation between the two [63]. Advances in our understanding of normal-appearing brain tissue damage, clinically-silent spinal cord damage and atrophy, grey matter and retinal nerve fiber layer involvement, cognitive impairment, and cortical adaptation in MS have helped explain some of the confounders to the clinical-radiological association. MRI lesion activity measures have not been accepted as validated surrogates because such measures lack the pathologic specificity for the processes that contribute to disability in MS. As illustrated in Figure 1(c), the relationship between a first attack or relapsing disease and the clinical outcomes in MS is complex. MRI measures alone have failed as surrogate markers of disability since other mechanisms for tissue injury exist beyond the inflammatory lesions that are visualized on MRI scans.

### 3.2. Optical Coherence Tomography

Optical coherence tomography (OCT) is an imaging modality which uses near infrared light to measure thickness and volumes of structures in the eye. Of particular interest is the retinal nerve fiber layer (RNFL), which consists of nonmyelinated axons, and macular volume, which comprises the axons and ganglion cell bodies. By quantifying the RNFL layer thickness and macular volume, OCT noninvasively captures anterior visual pathway axonal loss [64–66].

RNFL thickness has been proposed as a structural biomarker or surrogate measure for regional (optic nerve) and global (whole brain) axonal loss in MS [67]. In adults with MS, RNFL thickness is decreased compared to healthy controls by 5 um to 40 um on average, with greater thinning observed in the eyes of patients who have had optic neuritis [65, 67–69]. Longitudinal studies have also shown RNFL thinning in MS patients over time in the absence of clinical optic neuritis [70, 71]. RNFL thinning in adults with MS who have not had a clinical history or radiographic evidence of optic neuritis suggests that OCT also captures global brain atrophy in adults rather than local effects from optic nerve damage. The ability of OCT measures to inform upon global axonal injury in adult MS is further supported by the relationship between RNFL thickness and macular volumes with brain parenchymal fraction (BPF). In a study of 44 patients with a clinically isolated syndrome or multiple sclerosis, Young et al. found that RNFL thickness and total macular volume (TMV) as measured by OCT were both significantly associated with BPF as measured by MRI (*P* = 0.005 and *P* = 0.034 for RNFL thickness and TMV, resp.) [72]. Zimmerman et al. also found RNFL and TMV to be associated with white matter volume and normalized brain volume (*P* < 0.002 for both); however, contrary to Young et al, they also found RNFL thickness and TMV to be associated with grey matter volume (*P* ≤ 0.001 for both). Furthermore, Zimmerman et al. found these associations to be significant regardless of ON history [73].

Despite these data and further studies showing correlations between OCT measures, functional vision scores, quality of life scales, and the EDSS, RNFL thickness has not been validated as a surrogate for disability or disease progression in Phase 3 trials given the complex pathobiology in MS [15]. Pathologic changes in axons occur early in the disease and are prominent during the acute and progressive stages of MS [74]. Similar to the complexities using MRI metrics as surrogate outcome measures, OCT does not capture demyelination, and other pathways exist between the disease (either clinically isolated syndrome or relapsing-remitting MS) and the outcomes (disability, conversion to CDMS, or relapses). Nevertheless, OCT is still a promising tool for future trials, especially studies evaluating the effects of neuroprotective agents [50]. During an international meeting of selected experts (“Imaging outcomes for protection and repair in multiple sclerosis,” Amsterdam, The Netherlands, August, 2008), Barkhof et al. concluded that OCT fulfilled the five criteria for outcome measures in such trials: (1) pathologic specificity; (2) reproducibility; (3) sensitivity to change; (4) clinical relevance; and (5) response to treatment [50].

## 4. Pediatric MS Clinical Trials

Currently, disease-modifying therapies have not been approved for pediatric MS as clinical trials have not been performed in children less than 18 years. However, interferons, glatiramer acetate, natalizumab, and other therapies are prescribed off-label in the pediatric population based on adult trials and pediatric observational studies [75, 76]. While these therapies have been monitored for adverse effects and tolerability in children, additional data are needed, especially with respect to the risk of progressive multifocal leukoencephalopathy and exposure to JC virus. With respect to the newer agents approved for MS, such as fingolimod, teriflunomide, and dimethyl fumarate, long-term effects on neurological development and the maturation of a child's immune, endocrine, and reproductive systems, and neurological development are unknown [36]. Therefore, clinical trials in pediatric MS are needed and are currently underway given the recent EMA and FDA mandate that all newly approved drugs must include a pediatric investigation plan.

The conduct of clinical trials in pediatric MS will be dependent on the use of sensitive clinical outcome measures that capture the most relevant dimensions of function and disability. While the clinical symptoms of MS are similar in children and adults, there are notable differences in the relapse rate, disease burden on MRI, and disability based on the age at diagnosis. Some of these differences include the following.

- Children have a higher number of relapses in the first 2–5 years of disease compared to adults [77, 78].
- Children generally present with a second attack within 12 months of the first attack, although younger children may have a longer interval [79]. Adults generally take about 2–2.5 years to convert to clinically-definite MS [80, 81].
- Children have an increased T2 lesion burden in the brain at disease onset on MRI scans and more gadolinium enhancing foci [16, 78].
- Children, especially prepubertal, are more likely to initially have large ill-defined T2 lesions that resolve on follow-up scans and then develop focal ovoid lesions typical of adult-onset MS. In contrast, adults have focal ovoid T2 lesions that do not typically resolve [16, 78].

The EDSS may not be as sensitive in pediatric subjects compared to adults with MS due to a lesser degree of physical disability in children with MS. However, cognitive deficits have been recognized in pediatric MS subjects. Cognitive impairment occurs in approximately 30% of children with MS [53, 82]. Patient IQ is also significantly reduced in pediatric-onset MS, and lower IQ scores are associated with younger age at onset of disease [82]. Till et al. found decreased cognitive function to be associated with reduced thalamic and global brain volumes. Since T1 and T2 lesional volumes were not strong predictors of cognitive impairment, the authors concluded that neurodegenerative processes, rather than inflammation and relapses, are responsible for brain atrophy early in pediatric MS [53].

The differences between pediatric and adults MS and selection of outcome measures were considered by an expert panel of physicians, pharmaceutical representatives, and regulatory agencies while discussing the future of pediatric trials. The Steering Committee of the International Pediatric MS Study Group [36] agreed that annual relapse rate or time to next relapse should be used as the primary outcome measure for clinical trials but acknowledges that this may not always be feasible due to time constraints and cost. The Steering Committee of the International Pediatric MS Study Group also recommended several measures of cognitive function, which examine attention, functioning, verbal learning and memory, language, and general intelligence [36]. Sample size calculations for clinical trials of pediatric-onset MS have recently been proposed and may aid in planning phase II and III trials powered on clinical and MRI endpoints [83].

The comparison group for pediatric MS trials has also been debated. The inclusion of a placebo comparison group may be considered unethical since the disease-modifying therapies are generally considered safe and well-tolerated in children. An alternative approach is to use an interferon or glatiramer actetate for the control group when comparing a newer agent or novel treatment [84]. The latter would require a larger sample size, and the conduct of pediatric MS clinical trials has been further challenged by the low prevalence of disease in children.

## 5. Conclusion

Multiple sclerosis is a disabling disease and current treatment is aimed at slowing disease progression. Several outcome measures have been used in recent relapsing-remitting MS clinical trials to quantify clinical disease activity (relapses), MRI disease burden (lesion counts, lesion and brain volumes), and neurodegeneration (OCT). The selection of the outcome measures and the results of these trials should be interpreted with an understanding of the following. (1) MS is a heterogeneous disease and inclusion criteria should be carefully reviewed when results are applied to individual patients. (2) MS evolves over time from a predominantly inflammatory disease to a secondary progressive or neurogenerative process. As such, a clinical outcome measure may have different efficacy in the early stages of the disease compared to the advanced stages. (3) A surrogate may seem to indicate a positive correlation with the intervention, but the relationship could be attributed to other mechanisms. If the measure is not something that is in the causal pathway for MS, it may mislead researchers about the efficacy of the treatment or progression of the disease [1]. (4) The conduct of pediatric MS trials presents additional challenges that are being carefully reviewed and considered in the design of future studies. The combination of multiple endpoints in clinical trials has led to the successful approval of MS therapies for relapsing-remitting MS resulting in decreased disease burden, morbidity, and disability. Future trials, both adult and pediatric, will likely continue to use a combination of outcome measures to determine the efficacy of investigational therapies in slowing disease progression and modifying disability.

## Figures and Tables

**Table 1:** Outcome measures selected for clinical trials in relapsing-remitting multiple sclerosis.

Study name/group	Drug treatment	*N*	Publication year	Primary outcome	Secondary outcomes
Multiple sclerosis collaborative research group	Interferon beta-1a (avonex)	301	1990	Worsening in disability defined as deterioration by 1.0 point on EDSS	(i) Time to first relapse (ii) Number and volume of gadolinium enhancing lesions

Interferon beta-1b multiple sclerosis study group	Interferon beta-1b (Betaseron)	372	1993	(i) Annualized relapse rate (ii) Proportion of relapse-free patients	(i) Time to first relapse (ii) Relapse duration and severity by NRS score (iii) Change in EDSS and NRS scores from baseline (iv) Quantitative disease burden by MRI (% change in lesion area) (v) Disease activity as measured by MRI (annual rate of new or enlarging lesions)

Copolymer 1 multiple sclerosis study group	Copolymer 1 (glatiramer acetate, Copaxone)	251	1995	Average number of relapses at 2 years	(i) Proportion of relapse free patients (ii) Time to first relapse (iii) Proportion without sustained disease progression as measured by the EDSS at 3 months (iv) Average change in EDSS and ambulation index

PRISMS	Interferon beta 1a (Rebif)	560	1998	Number of relapses	(i) Times to first and second relapse (ii) Progression of disability as measured by sustained worsening of EDSS, ambulation index, or arm function index at 3 months (iii) Proportion of relapse-free patients (iv) MRI burden (proton density T2 MRI, Number of T2 active lesions)

CHAMPS	Interferon beta 1a (Avonex)	383	2000	Conversion to CDMS	(i) Volume of T2 lesions (ii) Number of new or enlarging T2 lesions (iii) Number of gadolinium enhancing T1 lesions

ETOMS	Interferon beta 1a (Rebif)	309	2001	Conversion to CDMS	(i) Change in the SNRS score (ii) Time to second relapse (iii) Number of new or enhancing T2 lesions (iv) Number of enhancing T1 lesions (v) Number of patients without MRI activity (no new or enlarging T2 lesions or enhancing lesions throughout the study) (vi) Yearly changes of hyperintense T2 lesion volume

EVIDENCE	Interferon beta-1a (Rebif)	677	2005	(i) Annualized relapse rate; (ii) Proportion remaining relapse free at 2 years	(i) Change in new, enlarged, or reappearing lesions (ii) Proportion of active scans per patient (new, enlarged, or reappearing lesions)

BENEFIT	Interferon beta-1b (Betaseron)	487	2006	(i) Time to conversion to CDMS; (ii) Time to McDonald defined MS	(i) Cumulative number of new or enhancing T2 lesions (ii) Change in T2 lesion volume (iii) Cumulative volume of gadolinium enhancing lesions (iv) Change in EDSS and MSFC scores

CHAMPIONS	Interferon beta 1a (Avonex)	203	2006	Conversion to CDMS	(i) Number of confirmed relapses (ii) Disease course classification (iii) Neurological disability as measured by EDSS at 5 years (iv) Number of new or enlarging T2 lesions (v) Changes in T2 volume (vi) Percentage with gadolinium enhancing lesions

AFFIRM	Natalizumab (Tysabri)	856	2006	(i) Annualized relapse rate; (ii) Progression of disability as measured by sustained worsening of EDSS at 12 weeks	(i) Number of new or enlarging T2 lesions (Year 1) (ii) Number of gadolinium enhancing lesions (Year 1) (iii) Proportion of relapse free patients (Year 1) (iv) Annualized relapse rate (Year 2) (v) Volume of lesions (Year 2) (vi) Number of new hypointense lesions (Year 2) (vii) MSFC as measure of disability (Year 2)

SENTINEL	Natalizumab and Interferon beta-1a (Tysabri and Avonex)	1003	2006	(i) Annualized relapse rate; (ii) Progression of disability as measured by sustained worsening of EDSS at 12 weeks	(i) Number of new or enlarging T2 lesions (Year 1) (ii) Number of gadolinium enhancing lesions (Year 1) (iii) Proportion of relapse free patients (Year 1) (iv) Annualized relapse rate (Year 2) (v) Volume of lesions (Year 2) (vi) Number of new hypointense T1 lesions (Year 2) (vii) MSFC as measure of disability (Year 2)

REGARD	Interferon beta-1a and Glatiramer acetate (Rebif and Copaxone)	764	2008	Time to relapse at 96 weeks	(i) Number of new or enhancing lesions (ii) Number of gadolinium enhancing lesions (iii) Change in T2 lesion volume (iv) Change in whole brain volume (v) Other relapse outcomes (vi) Progression of disability as measured by sustained worsening of EDSS at 6 months

BECOME	Interferon beta-1b and glatiramer acetate (Betaseron and Copaxone)	75	2009	Number of combined active lesions in the first year (total contrasting enhancing lesions plus new non-enhancing lesions that have appeared since most recent examination)	New lesions per subject in year 1 and 2

PreCISe	Glatiramer acetate (Copaxone)	619	2009	Time to conversion to CDMS	(i) Number of new T2 lesions (ii) Baseline-adjusted T2 lesions volume at last scan (iii) Brain atrophy- percentage change from baseline (iv) Proportion of patients converted to MS

TRANSFORMS	Fingolimod (Gilenya)	1153	2010	Annualized relapse rate	(i) Number of new or enlarged T2 hypointense lesions at 1 year (ii) Time to confirmed disability progression as measured by EDSS at 3 months

FREEDOMS	Fingolimod (Gilenya)	1272	2010	Annualized relapse rate accompanied by change in EDSS	(i) Time to confirmed disability progression as measured by EDSS at 3 months (ii) Time to first relapse and disability progression at 6 months (iii) Change in EDSS and MSFC at 2 years (iv) Number of patients with gadolinium enhancing lesions (v) Number with new or enlarging T2 weighted lesions (vi) Proportion of patients with gadolinium-enhancing lesions and T2-weighted lesions (vii) T2 and T1 lesion volume (viii) Change in brain volume at 2 years

TEMSO	Teriflunomide (Aubagio)	1088	2011	Annualized relapse rate	(i) Progression of disability as measured by EDSS at 12 weeks (ii) Total lesion volume (iii) Volume of hypointense T1 lesions (iv) Number of gadolinium enhanced T1 lesions (v) Number of active lesions (new gadolinium enhancing on T1 images or new or enlarged lesions on T2) (vi) Brain parenchymal fraction

DEFINE	BG-12 (dimethyl fumarate, Tecfidera)	1237	2012	Proportion of patients who had a relapse at 2 years	(i) Number of gadolinium enhancing lesions (ii) New or enlarged hyperintense T2 lesions (iii) Annualized relapse rate (iv) Time to progression of disability as measured by EDSS at 12 weeks

CONFIRM	BG-12 and glatiramer acetate (Tecfidera, Copaxone)	308	2012	Annualized relapse rate at 2 years	(i) New or enlarging hyperintense lesions on T2 (ii) Number of hypointense T1 lesions (iii) Proportion of patients with relapse (iv) Time to disability progression as measured by EDSS at 2 years

PRISMS: Prevention of Relapses and Disability by Interferon beta-1a Subcutaneously in Multiple Sclerosis Study; CHAMPS: Controlled High Risk Avonex Multiple Sclerosis Study; ETOMS: Early Treatment of Multiple Sclerosis Study Group; EVIDENCE: Evidence of Interferon Dose-Response: European North American Comparative Efficacy; BENEFIT: Betaferon in Newly Emerging Multiple Sclerosis for Initial Treatment; CHAMPIONS: Controlled High Risk Avonex Multiple Sclerosis Prevention Study In Ongoing Neurological Surveillance; AFFIRM: Natalizumab Safety and Efficacy in Relapsing Remitting Multiple Sclerosis; SENTINEL: The Safety and Efficacy of Natalizumab in Combination with Interferon Beta-1a in Patients with Relapsing Remitting Multiple Sclerosis; REGARD: Rebif versus Glatiramer Acetate in Relapsing MS Disease; BECOME: Betaseron versus Copaxone in Multiple Sclerosis with Triple-Dose Gadolinium and 3-Tesla MRI Endpoints; PreCISe: Effect of glatiramer acetate on conversion to clinically definite multiple sclerosis in patients with clinically isolated syndrome; TRANSFORMS: Trial Assessing Injectable Interferon versus FTY720 Oral in Relapsing-Remitting Multiple Sclerosis; FREEDOMS: FTY720 Research Evaluating Effects of Daily Oral Therapy in Multiple Sclerosis; TEMSO: Teriflunomide Multiple Sclerosis Oral; DEFINE: Determination of the Efficacy and Safety of Oral Fumarate in Relapsing-Remitting Multiple Sclerosis; CONFIRM: Comparator and an Oral Fumarate in Relapsing-Remitting Multiple Sclerosis.

EDSS: Extended Disability Status Scale; MSFC: Multiple Sclerosis Functional Composite; CDMS: Clinically Definite Multiple Sclerosis; SNRS: Scripp's Neurological Rating Scale; NRS: Neurological Rating Scale.
